# Origin of replication discovery for environmentally isolated *Pantoea* strain enables expression of heterologous proteins, pathways and products

**DOI:** 10.1016/j.isci.2026.115031

**Published:** 2026-02-17

**Authors:** Alex Codik, Ankita Kothari, Hualan Liu, Benjamin L. Weinberg, Trenton K. Owens, Aparajitha Srinivasan, Alex Rivier, Thomas Eng, Adam P. Arkin, Adam M. Deutschbauer, Aindrila Mukhopadhyay

**Affiliations:** 1 Biological Systems and Engineering Division, Lawrence Berkeley National Laboratory, Berkeley, CA 94720, USA; 2 Comparative Biochemistry Graduate Group, University of California, Berkeley, Berkeley, CA 94720, USA; 3 US Department of Energy Joint Genome Institute, Lawrence Berkeley National Laboratory, Berkeley, CA 94720, USA; 4 Environmental Genomics and Systems Biology Division, Lawrence Berkeley National Laboratory, Berkeley, CA 94720, USA; 5 Department of Plant and Microbial Biology, University of California, Berkeley, Berkeley, CA 94720, USA; 6 Department of Bioengineering, University of California, Berkeley, Berkeley, CA 94720, USA

**Keywords:** molecular biology, molecular genetics, molecular mechanism of gene regulation, molecular microbiology

## Abstract

Leveraging predicted origin sequences from a previously characterized groundwater plasmidome, we constructed a barcoded plasmid library to screen for previously unknown origins. Testing this library against a panel of representative bacterial strains led to the identification of 3 previously unknown origins that replicate in gram-negative bacteria not previously associated with these origin sequences. Experimental validation confirmed that a plasmid bearing origin 6911 as the sole origin could replicate with a copy number of 9 (±2) in *Pantoea* sp. MT58, a fast growing and metal tolerant, environmentally important bacterium. Plasmids based on this new origin were used to express the reporter protein GFP, and non-native metabolite pathways for the natural product indigoidine and the terpenoid compound isoprenol. Functional previously unknown origins of replication in such non-model organisms can expand the toolkit for genetic manipulations of both model and less-studied bacteria.

## Highlights

• Plasmid library constructed with known and predicted bacterial origins of replication
• Discovery of 3 previously unknown origins that replicate in gram-negative bacteria
• Origin validation in *Pantoea* sp. MT58 through copy number determination
• Bioproduction of GFP, indigoidine, and isoprenol in *Pantoea* sp. MT58

## Graphical abstract

**Figure:**
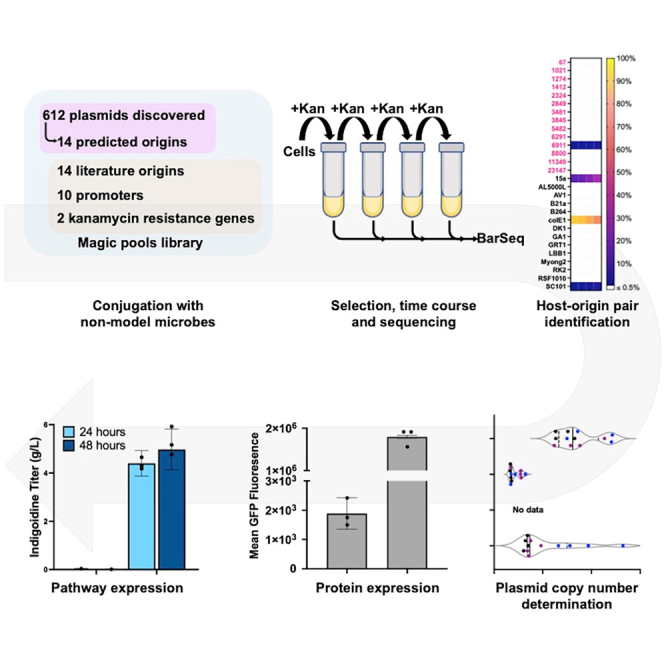

## Introduction

Genetic transformation is a critical step to transition from a genomics-based examination of a bacterial strain to genetics. Transformation of a plasmid capable of replicating in a bacterial host enables an array of genetic approaches that are indispensable in characterizing or engineering microbial strains. In spite of this, it can be a challenge to find and/or construct a plasmid that is able to self-replicate in a microbe of interest.^1^^,^^2^^,^^3^^,^^4^^,^^5^ Specifically, the plasmid’s origin of replication is a key requirement but predicting origins that will function in a given host is challenging.^6^ However, discovery of new origins that function in diverse, non-model microbes has not been extensively explored.

Recent advances have allowed for the identification of many features of plasmids that are valuable in understanding the functional role of a plasmid as a mobile genetic element in the environment,^7^^,^^8^^,^^9^^,^^10^^,^^11^^,^^12^ and to reveal biological parts that can be used in molecular biology approaches.^11^^,^^12^^,^^13^^,^^14^^,^^15^ However, identification of the sequence that encodes the origin of replication is challenging and design of origins *de novo* remains elusive. One path forward is the use of existing plasmid sequences that can be analyzed to predict putative origin sequences and can then be experimentally verified. A plasmidome is the total collection of bulk plasmids present in a given environment that can be measured by whole genome sequencing (WGS).^16^ Targeted plasmidome studies, as well as metagenomics studies, reveal a vast range of native plasmids that provide a deep resource to address this challenge.^17^^,^^18^^,^^19^^,^^20^

In this report, we leveraged a previously characterized plasmidome from groundwater samples from the Oak Ridge field research center (ORFRC) containing over 600 unique plasmid sequences.^17^ This prior plasmidome dataset revealed key aspects of both the nature and the function of these mobile genetic elements, which included both plasmid^17^ and viral sequences.^21^ Earlier studies focused on key features of the native plasmid scaffolds such as the range of mobilization mechanisms and incompatibility groups that are spanned by these plasmids, and the functions annotated on these sequences. One study fully synthesized a native plasmid and tested functions and host ranges experimentally.^22^ One aspect that remained unexplored was use of these putative plasmid scaffolds for parts discovery. Specifically, we aimed to obtain the origin of replication sequences that could enable us to build genetic tools for new and existing microbial systems. Thus in this study we selected a subset of the groundwater plasmidome sequences from the published plasmidome data and analyzed them for putative predicted origins of replication.

A primary goal of our study was to establish a workflow that would experimentally validate predicted origin of replication sequences, enable identification of new host-plasmid pairings and advance the usable genetic tools in new environmental and biotechnologically important bacterial strains. To do this, we adopted a magic pool approach to build a DNA barcoded plasmid library containing the predicted origin sequences.^23^ A magic pool is a DNA-barcoded plasmid library with different genetic parts, such as promoters and drug resistance genes, which can be used to quickly find a suitable combination of parts for a target bacterium.^23^ We experimentally screened the plasmid pool containing a combination of putative and known origins against a well-studied model bacteria (e.g., *E. coli* BW25113) and less-studied environmental strains (e.g., *Pantoea* sp. MT58). The *Pantoea* genus is especially important for further studies; it is a widely distributed genus found both in plant and soil associated communities spanning pathogenic^24^^,^^25^^,^^26^^,^^27^ and commensal^25^^,^^28^ species. *Pantoea* sp. are also seen as a valuable new platform for biotechnology applications.^14^^,^^25^^,^^29^^,^^30^^,^^31^ In both of these contexts deeper genetic tools and an available model-system, such as the Gammaproteobacteria *Pantoea* sp. MT58 could be valuable. In the ORFRC, *Pantoea* sp. MT58 is a member of the sediment microbial community that is gaining interest due to its metal tolerance capabilities,^32^ and a recent pangenome study for this bacterium highlighted its facultative anaerobic capabilities being involved in survival in the contaminated groundwater at the ORFRC.^33^ In our study we sought to discover additional genetic parts for use in this emerging microbe of interest and demonstrate the use of new plasmids for expression of proteins and pathways.

## Results

### Environmental plasmids used to predict origins of replication

**Figure 1:**
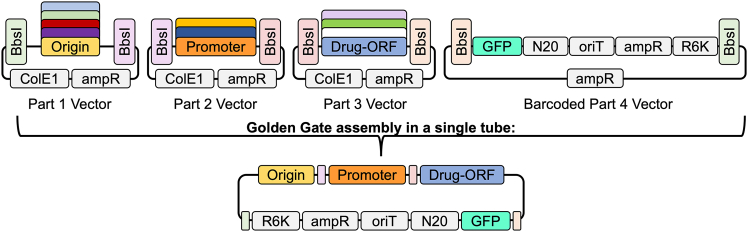
Magic pool construction method Each colored BsbI box represents a unique sticky end cut site that will complement only the BsbI boxes with the same color to link plasmid parts together sequentially. N20 is representative of the 20 base pair barcodes.

While there are robust resources for plasmid discovery and analysis, prediction of plasmid origins of replication sequences has fewer tools.^7^^,^^34^^,^^35^ DoriC, originally developed as a database for *oriC* prediction of bacterial and archaeal chromosomes, was updated with plasmid sequences in DoriC 10.0.^36^ Thirty two sub-selected circular plasmids from our earlier plasmidome study^17^ were assessed using this tool and led to 18 predicted plasmid-based origins of replication that could then be experimentally tested. These 18 origin of replication sequences were synthesized as double strand DNA fragments (see STAR Methods) on a backbone compatible with the magic pool library construction method (Figure 1). We also examined the 32 native plasmid scaffolds in a recently published plasmid oriV predictor, OriV-finder.^37^ The majority of the origins of replication in the initially predicted set were also predicted via this tool, along with additional oriV sequences (Data S1). Notably, while naturally occurring plasmids may have multiple origins of replication or replication machinery, these workflows predicted at most one origin for each plasmid.

### Origins magic pool library constructed to test origin-host pairings

A magic pool library was constructed using 4 different parts, as outlined in Figure 1. The magic pool was constructed using 38 variants of origins of replication (part 1), 10 variants of kanamycin drug resistance gene promoters (including ribosomal binding site [RBS]) (part 2), and 2 variants of kanamycin drug resistance genes (part 3), per the original design of the magic pools plasmid scaffold.^23^ Variants of parts 2 and 3 were chosen to ensure effective kanamycin selection in the target microbe. The construction of this library was performed using golden gate assembly and was sequenced using long-read sequencing. In the mapping of the barcode-part association of the magic pool it was found that the colE1-associated plasmids were disproportionately abundant, likely due the high copy number of this origin in *E. coli*.

In the library mentioned above, part 1 is the variable part; the origin sequence and the key component being assessed, represented by one of 18 putative origins or one of 20 origins mined from literature (Data S1). Parts 2 and 3 came from a previous study.^23^ Part 4 was the barcoded backbone for all plasmids constructed in the magic pools and contains a conditional R6K origin of replication for plasmid maintenance in *E. coli*, an origin of transfer, and an antibiotic selection marker (carbenicillin). The DNA barcodes are random 20 nucleotide sequences flanked by common PCR priming sites. The barcodes are introduced into the part 4 plasmid backbone via golden gate assembly from a PCR product containing the random 20 mers, as previously described in Liu et al.^23^ The sequences of parts 1, 2, and 3 can be found in Data S1.

The magic pool backbone (part 4) has a barcode which can establish a plasmid-barcode association. These associations consist of a single barcode and a combination of parts (a single origin, a single promoter, and a single kanamycin drug resistance gene). Barcodes that linked to multiple plasmids or combinations of parts were eliminated from consideration. This resulted in 8,802 total barcoded plasmids that mapped a unique barcode to a part 1 origin (some of these barcodes could not be confidently assigned a part 2 or part 3). The number of barcodes associated with each origin in the magic pool can be seen in Figure S1.

### Barcode sequencing reveals host-origin pairings

**Figure 2:**
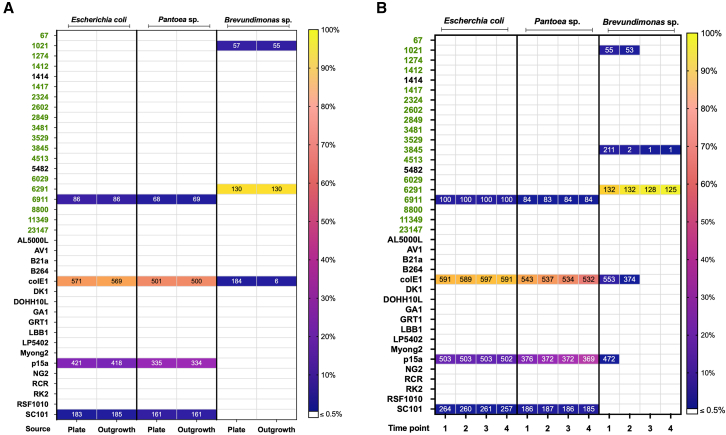
Magic pool BarSeq results for all origin sequences tested The y axis denotes the names of the origins of replication, with predicted origins in green, and origins found in literature in black. Colors in each time point, or source, square represent the fraction of barcode counts associated with that origin versus the total fraction of barcodes in that sample (see legend). The number present in each box represents the total number of unique barcodes detected for that particular origin of replication at that time point, or for that source. (A) Plate-based sources for the magic pool conjugations with the 3 hosts. “Plate” is indicative of cells harvested directly from an agar plate, while “Outgrowth” is indicative of an overnight of growth in liquid media after scraping colonies off of an agar plate. (B) Liquid-based time points for magic pool conjugations with the 3 hosts. Time points 1, 2, 3 and 4 represent 24, 48, 72, and 96 h respectively. Note that the recipient strain *E. coli* BW25113 is part of the recipient set along with *Pantoea* MT58 and *Brevundimonas sp*, and is different from the conjugation strain *E. coli* WM3064, which was used as the donor. Note also that the 3845 hit for *Brevundimonas* sp. was analyzed due to a single bar code enrichment and represents an artifact rather than a real candidate.

To evaluate the compatibility of the various plasmid origins across different bacterial species, the magic pool library was introduced into three distinct hosts: *Escherichia coli* BW25113,^38^ *Pantoea* sp. MT58,^32^ and *Brevundimonas* sp. GW460-12-10-14-LB2^23^ (Data S2: strain and plasmid table). We then used barcode sequencing of the resulting bacterial populations to quantify the relative enrichment of each plasmid variant, which allowed us to infer the origin compatibility in each host. To determine whether different selection strategies influenced the plasmid populations recovered, we applied two complementary approaches: plate-based selection and serial liquid outgrowth. In both workflows, comparison of barcode abundances against the map of the pooled library enabled us to determine which plasmid variants were retained under each selection condition (Figure 2). Similarly, we analyzed the data for high-confidence part 2 and part 3 sequences that worked effectively for driving kanamycin resistance in each strain (see Figures S2–S4 for part 2 and 3 variants breakdown in all strains).

Across both selection methods *E. coli* BW25113 and *Pantoea* sp. MT58 showed enrichment of four origins (Figure 2A). Three of these were established origins namely p15a, colE1, and SC101 all of which were previously shown to enable plasmid replication in *E. coli*.^39^^,^^40^^,^^41^ The previously uncharacterized 6911 origin showed enrichment in both *E. coli* BW25113 and *Pantoea* sp. MT58.

In *Brevundimonas* sp. GW460-12-10-14-LB2 two previously unknown origins—1021 and 6291 were enriched in plate-based selection. We detected barcode counts from colE1-containing plasmids in this strain, but we do not consider this a functioning origin by our analysis, because only a fraction of the colE1-associated barcodes was detected.

On the other hand, during serial liquid passaging, plasmids carrying the 6291 origin outcompeted all others and dominated the *Brevundimonas* population by the final time point. This outcome is consistent with selective dynamics in liquid culture, where origins with higher copy numbers can lead to stronger kanamycin resistance and thus confer a fitness advantage. The disproportionate enrichment of 6291 likely masked the 1021 origin signal, even though 1021 was functional in the plate-based method. Barcode counts attributed to origins 3845, colE1, and p15a were low and declined over time, consistent with carryover from the *E. coli* donor strain. In support of this, these are all high-abundance parts in the magic pool (Figures S1 and S4).

Together, these results demonstrate that the pooled library screening reliably identifies plasmid origins capable of replication in diverse bacteria and highlight host-specific differences in origin compatibility and competitive dynamics. Of note, this approach uncovered previously uncharacterized origin 6911 as broadly functional in both *E. coli* BW25115 and *Pantoea* sp. MT58.

### Presence of two origins of replication changes the plasmid copy number in *Pantoea* sp. MT58

Many magic pool generated plasmids containing the previously unknown origin 6911 showed evidence of driving plasmid replication in *Pantoea* sp. MT58 and *E. coli* BW25113, based on the BarSeq results (Figure 2). We further investigated this origin of replication and its copy number in the environmentally isolated strain *Pantoea* sp. MT58.

**Figure 3:**
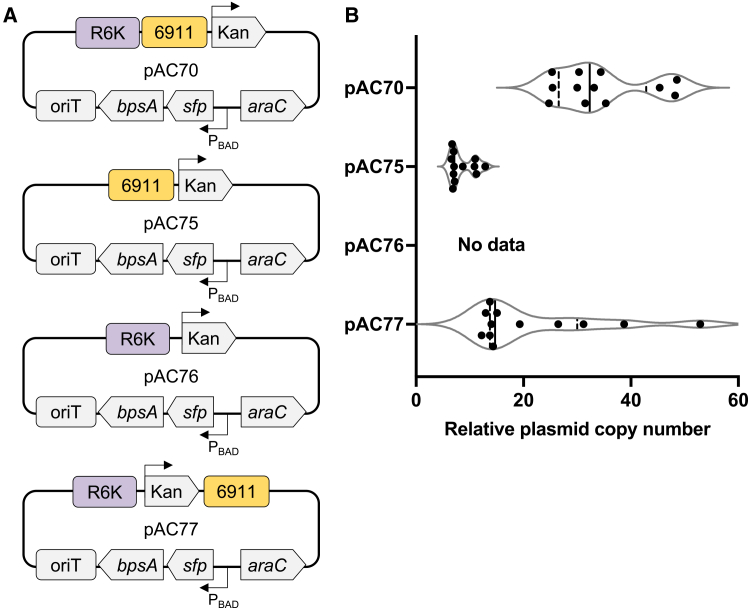
Validation of previously unknown origin 6911 in *Pantoea* sp. MT58 (A) Plasmid maps that correspond to the plasmids being tested in (B). (B) Violin plot of plasmid copy number. Data for each plasmid is from 3 biological replicates at 4 dilutions. A solid vertical line represents the data median. Vertical dashed lines represent quartiles.

The backbone of the magic pool contains an R6K origin for cloning and for maintaining all of the plasmids in *E. coli* pir+ strains. The existence of this origin of replication in the backbone of a magic pool used to test origins of replication requires the decoupling of the R6K from the origin being tested in the host-origin pair. To decouple the R6K present in the magic pool backbone and the origin 6911, and to test the use of this origin, four plasmids were constructed (Figure 3A). These four plasmids were constructed to contain the parts from the magic pool that were necessary (6911 origin and/or conditional R6K origin, kanamycin promoter and resistance gene, and oriT) but also contained a small two-gene pathway to more accurately represent a real world use and application of this plasmid.

The first plasmid was identical to the plasmid found in the library in regards to the conditional R6K origin and the origin 6911 (Figure 3A, plasmid pAC70). This plasmid contains the R6K origin proximal to the origin 6911. The second plasmid constructed contains only the origin 6911 as a replication mechanism (Figure 3A, plasmid pAC75). The third plasmid contains only the R6K origin as a replication mechanism (Figure 3A, plasmid pAC76). The final plasmid was constructed with both origins, R6K and 6911, but has placed these origins distal from each other, to see if R6K affects origin 6911 even when not proximal (Figure 3A, plasmid pAC77).

All four plasmids were individually conjugated into *Pantoea* sp. MT58 and successful conjugations were verified by the appearance of kanamycin resistant colonies whereas the control sample did not exhibit spontaneous kanamycin resistance. All plasmids, except for pAC76, were successfully conjugated into *Pantoea* sp. MT58. This indicates that R6K alone was not sufficient for plasmid replication but origin 6911 alone was sufficient for plasmid replication in *Pantoea* sp. MT58.

Transformants were then examined using colony-based quantitative PCR (qPCR) to obtain plasmid copy number estimates. qPCR was used to compare the genomic DNA amplicon relative to the plasmid amplicon to provide the number of plasmid copies per genome copy, a relative plasmid copy number per cell, for each plasmid. Rounded to the nearest whole number, pAC70 had a relative copy number of 34 (±9), pAC75 had a relative copy number of 9 (±2) and pAC77 had a relative copy number of 22 (±13) (Figure 3B and Data S3). The method was also tested for a plasmid containing colE1, reported to have 25–30 copies per cell.^41^ Colony-qPCR determined that, in *E. coli* 10-β, the plasmid had a copy number of 38 (±11) (Figure S5). While this experiment queried only origin 6911, the data suggests that having an origin of replication on a plasmid that also contains an R6K origin of replication may affect the plasmid copy number and the plasmid’s replicative capacity in a host.

### Expressing a GFP reporter using the previously unknown origin 6911 in *Pantoea* sp. MT58

**Figure 4:**
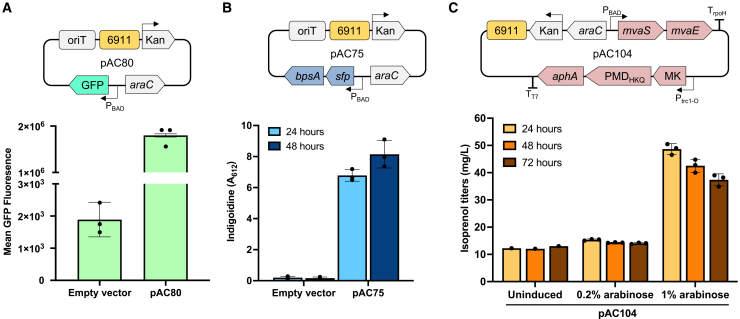
Applications of origin 6911 in *Pantoea* sp. MT58 (A) *Pantoea* sp. MT58 GFP expression (bottom panel) using plasmid containing only origin 6911 (top panel). (B) Indigoidine absorbance measurements from expression in *Pantoea* sp. MT58 (bottom panel) using a plasmid containing only origin 6911 (top panel). (C) Isoprenol production levels at different inducer levels (bottom panel) using a 6911-containing plasmid (top panel). Error bars in all three panels represent standard deviation from three biological replicates.

The backbone of each plasmid in the magic pool encodes a green fluorescent protein (GFP), a commonly used reporter protein in synthetic biology workflows in multiple microbial systems.^42^^,^^43^ In our study, GFP can be used to test functional origins of replication as well as expression of a heterologous protein in a new host. The original plasmid for *Pantoea* sp. MT58 did not show any GFP expression. However, introduction of an inducible promoter, P_BAD_, (Figure 4A), led to expression of GFP (Figure 4A). Representative flow cytometry samples can be seen in Figure S5, and data can be seen in Data S4.

### Demonstrating the utility of the origin 6911 for plasmid-based heterologous pathway expression in *Pantoea* sp. MT58

The successful expression of a reporter gene, GFP, in *Pantoea* sp. MT58 using the origin 6911 containing plasmid pAC80 motivated us to further investigate the potential to express pathways for non-native and secondary metabolites. To this end, we transformed *Pantoea* sp. MT58 with arabinose inducible (P_BAD_) multi-gene pathway plasmids, pAC75 and pAC104 in parallel experiments and produced a non-native non-ribisomal peptide product indigoidine, and the hemiterpene compound isoprenol, respectively (Figure S7).

In the engineered *Pantoea* strain heterologously expressing *sfp*_*B. subtilis*_::*bpsA*_*S. lavendulae*_ on the plasmid pAC75, upon induction by 0.3% (w/v) arabinose and extraction of harvested cells with DMSO, we observed an average OD_612_ of 4.4 at 24 h and 5.0 at 48 h (Figure 4B and Data S5). The OD_612_ values are indirect measures of indigoidine titers and confirmed expression of the two-step indigoidine pathway containing the phosphopantetheinyl transferase (*sfp*) and the non-ribosomal peptide synthetase (NRPS, *bpsA*). Successful production of indigodine represents an ability to express a non-native natural product in *Pantoea* sp. MT58 leveraging the plasmid-based expression system enabled by the origin 6911. Indigoidine is also a valuable bio-based synthetic indigo alternative that has been previously reported in other industrially relevant hosts.^44^^,^^45^^,^^46^^,^^47^^,^^48^

We also validated the use of this previously unknown origin 6911 based plasmid to express products from more complex multi-gene pathways. For this we used the heterologous isoprenoid pathway for the hemiterpene short chain alcohol isoprenol. A previously reported plasmid pIY670^49^ was converted into pAC104 by swapping the origin RK2 (optimized for expression in *P. putida*) with the origin 6911. Terpenes span a wide array of compounds from those useful in altering the metabolic profile of a microbe to those that serve as bioproduction targets. In this case, isoprenol is also a useful commodity chemical and a well-established biofuel precursor.^49^^,^^50^ Short chain alcohols also serve important roles in fitness and interactions in microbial communities^51^^,^^52^^,^^53^^,^^54^ and an ability to express them provides additional tools to alter and examine microbial systems in context of their community. In our system, we observed a maximum isoprenol production of 48.67 mg/L in 24 h (Figure 4C and Data S6) in glucose minimal medium. A drop in isoprenol titers (to 37.38 mg/L) is observed by 72 h and consistent with published reports.^49^ *Pantoea* sp. MT58 cannot utilize isoprenol as the sole carbon source (Figure S8A), but this strain encodes many alcohol dehydrogenases^55^ that can degrade isoprenol. Hence, the decrease in titers at 72 h in the engineered *Pantoea* sp. MT58 could be attributed to either the inherent volatility of isoprenol and/or non-catabolic degradation.

Between the two non-native systems, inducer requirement was markedly higher to achieve metabolite production in the isoprenoid system (1% w/v arabinose) vs. that from the NRPS system for indigoidine (0.3% w/v arabinose) (Figures 4B and 4C). While indigoidine is produced from the amino acid precursor-glutamate, isoprenol is derived from acetyl-CoA which is a key central metabolite at the intersection of carbohydrate, lipid, and protein metabolism with higher competing pathway demand. Additionally, the heterologous isoprenol pathway is also cofactor-dependent^49^ compared to the indigoidine pathway which could impose a metabolic burden. The accumulation of pathway intermediates may impose additional toxicity even though *Pantoea* sp. MT58 is tolerant to up to 7 g/L of the final product-isoprenol (Figure S8B).^56^^,^^57^^,^^58^ Furthermore, *Pantoea* sp. MT58 consumes arabinose as a sole carbon source (Figure S9). These results establish this previously unknown origin 6911 as a promising genetic tool that improved the genetic tractability of *Pantoea* sp. MT58, and are an important first step in strain domestication to investigate the ecological role of this bacterium and to develop industrially relevant hosts. However additional optimizations are needed to use these systems to either modulate the levels of these metabolites to examine their role in community interactions, or to facilitate the development of chassis strains.

## Discussion

Discovering replicable plasmids is essential to moving from genomics-to genetics-based approaches. We leveraged a large plasmidome dataset from prior reports to specifically focus on plasmid origin sequences as these could be used to develop engineered replicating plasmids. It is interesting to consider that the native plasmid scaffolds used in the present study each have their own host range and plasmid replicating features. Our prior studies have explored the host range of these extrachromosomal genetic materials (including plasmids and viral genomes), both via direct assay of microbial hosts with synthesized plasmids^22^ and predicting hosts for viral genomes.^21^ However a non-native replicating plasmid containing only the sequence for such novel origins of replication may have a different host range. While newer prediction tools such as the OriV-finder^37^ corroborated the sequences selected for building the magic-pools library, the functional validation of both the hosts in which the resulting plasmid can operate, and its characteristics, need to be conducted experimentally. The OriV-finder tool predicted additional sequences relative to our original set, and new AI/ML tools in this domain^59^ may soon provide a large number of putative origin sequences. This further necessitates an experimental workflow as we have developed to empirically validate efficacy in a range of microbial hosts.

Our workflow identified a previously unknown origin for the non-model *Pantoea* sp. MT58, which was confirmed through qPCR experiments by decoupling of the conditional R6K origin and the origin 6911. The variability in copy number seen in the two plasmids that contain both the R6K and the 6911 origin may be due to the competing/commensal origins, a change in plasmid partitioning or replication mechanisms. This is clearly different from the 6911-only plasmid’s tight variability. Occasionally multi-origin plasmids are found in nature,^60^ and studying these interactions may help elucidate the specific interaction between the R6K and 6911. The low copy number origin 6911 was then used to express three molecules, each with increasing complexity, in *Pantoea* sp. MT58. While the magic pools library was used to screen a small subset of hosts, both model and non-model, it can be expanded to include additional bacterial hosts for further discovery. The products described in this study are highly relevant to key metabolic pathways, and the production of chemicals and fuels. Now that we have shown basic capabilities to express genes and pathways to produce heterologous proteins and metabolites, additional optimization of promoter strength, metabolic burden, or host physiology can further enhance production and lead to the use of this microbe as a bioproduction chassis. Overall the development of genetic tools for this microbial host has value in its use as a biotechnology platform as well as in future studies for understanding its role in the environment.

Discovery of novel origins of replication add to the existing genetic toolkit for bacteria, like *Pantoea* sp. MT58, but for others it could provide an entirely new opening for genetics, like in *Brevundimonas* sp. GW460-12-10-14-LB2. In this study we focused on validating previously unknown origin based plasmids in *Pantoea* sp. MT58 due to its importance as a denitrifying microbe in ground water and sediments systems^32^^,^^33^^,^^61^ as well as a representative microbe from the biologically important *Pantoea* genus.^24^^,^^25^^,^^26^^,^^27^^,^^28^ In our study we could also characterize important features of the new plasmid such as copy number. However, other aspects, such as the compatibility group, of these new plasmids remain uncharacterized. It is important to note that many aspects of plasmid function are dependent on the host, presence of other plasmids or replicating units and the functional genes encoded on them. As more plasmids become available for these relatively new genetically tractable bacterial systems, characterization of such features will also be possible. Exploiting these new tools in environmental bacteria requires additional investigation and experimentation, but promises to provide a range of new tools and approaches in many new microbial systems.

### Limitations of the study

While this study used DoriC to predict plasmid-based origins of replication, the tool itself is designed to work with genomes. However, recent development of plasmid databases and prediction tools has the potential to alleviate this issue. In our study we examined our native scaffold set using one of these recent tools, OriV-finder, and found useful additional origins of replication sequences that provide targets for future experimental validation.

The conditions under which a transformation occurs can greatly influence the success or efficiency, particularly antibiotic concentrations and media formulations. When transforming a pooled library of plasmids into bacterial strains, these conditions can cause false negative results. Without a significant effort to disprove these false negatives it becomes very difficult to distinguish a true negative and a false negative. As such, the positive results provide useful genetic tools, but the sequences with no transformation under the tested conditions could be tested in additional microbial hosts under alternate selection, recovery, and outgrowth conditions. However, if the goal is to discover plasmids or origins for a specific high-interest microbial target, many antibiotic conditions, and media formulations should be tested.

## Resource availability

### Lead contact

Requests for further information and resources should be directed to and will be fulfilled by the lead contact, Aindrila Mukhopadhyay (amukhopadhyay@lbl.gov).

### Materials availability

Materials will be made available upon request.

### Data and code availability

Data: All sequencing data has been deposited in NCBI and is publicly available as of the date of publication. Accession numbers are listed in the key resources table.

Code: This paper does not report original code. All software and algorithms used for analysis are listed in the key resources table.

Other: This paper does not report any additional resources.

## Acknowledgments

The authors thank the Mukhopadhyay group for their constructive feedback regarding the manuscript. Dr. Shweta Priya (LBNL) provided valuable guidance in conducting the qPCR experiments. We want to thank Dr. Guilherme de Siqueira (LBNL) for help in uploading sequencing data to NCBI. We thank the authors of DoriC and OriV-finder for help with assessing putative origins of replication. We also acknowledge the JBEI (jbei.org) strain collection for one of the strains used in this manuscript. This material is based upon work by the Ecosystems and Networks Integrated with Genes and Molecular Assemblies (ENIGMA) (http://enigma.lbl.gov) project, a Science Focus Area Program at Lawrence Berkeley National Laboratory (10.13039/100006235LBNL) supported by the U.S. Department of Energy, Office of Science, Biological and Environmental Research under Contract Number DE-AC02-05CH11231.

## Author contributions

A.C., A.K., and A.M. developed the study. A.K., H.L., and A.M.D. designed the origins magic pool library. A.C. and to build the library. A.C. and B.L.W. tested the library. A.C. performed qPCR, GFP and indigoidine experiments. A.R. developed electroporation methods for *Pantoea* sp. MT58. A.C. and A.S. performed isoprenol experiments, with A.S. performing GC-FID data analysis. A.C. performed all other data analysis. T.E., A.M.D., and A.M. provided supervisory roles and feedback. A.P.A., A.M.D., and A.M. acquired the funds for the project. A.C. drafted the initial manuscript. All authors have read, provided feedback, and approved the manuscript for publication.

## Declaration of interests

The authors declare no competing interests.

## STAR★Methods

### Key resources table

**Table:** 

REAGENT or RESOURCE	SOURCE	IDENTIFIER
**Bacterial strains**		

*Escherichia coli* BW25113	JBEI isolate collection	N/A
*Escherichia coli* TransforMax EC100D *pir*-116	Biosearch Technologies	Cat#EC100D
*Escherichia coli* WM3064	Biosearch Technologies	Custom order
10-beta Competent *Escherichia coli*	New England Biolabs	Cat#C3019H
*Pantoea* sp. MT58	ENIGMA isolate collection	N/A
*Brevundimonas* sp. GW460-12-10-14-LB2	ENIGMA isolate collection	N/A

**Chemicals, peptides and recombinant proteins**		

LB Broth, Miller	BD Difco	Cat#244620
Carbenicillin (100 mg/mL)	Teknova	Cat#C2135
Kanamycin (50 mg/mL)	Teknova	Cat#K2127
Diaminopimelic acid (DAP)	Sigma-Aldrich	Cat#D1377
NEBuilder HiFi DNA Assembly Master Mix	New England Biolabs	Cat#M5520AA
BpiI (BbsI) (10 U/μL)	Thermo Fisher Scientific	Cat#ER1011
Buffer G (10X)	Thermo Fisher Scientific	Cat#BG5
T4 DNA Ligase (2,000,000 U/mL)	New England Biolabs	Cat#M0202M
Sodium phosphate dibasic	Sigma-Aldrich	Cat#S9763
Potassium phosphate monobasic	Sigma-Aldrich	Cat#P0662
Sodium chloride	Sigma-Aldrich	Cat#S9888
Glucose solution	Teknova	Cat#G2020
Ammonium sulfate	Sigma-Aldrich	Cat#A4915
Magnesium sulfate	Sigma-Aldrich	Cat#208094
Calcium chloride	Sigma-Aldrich	Cat#C4901
Trace metal solution	Teknova	Cat#T1001
Arabinose	Sigma-Aldrich	Cat#A3256

**Critical commercial assays**		

QIAprep Spin Miniprep Kit	Qiagen	Cat#27106
Q5 High-Fidelity DNA Polymerase	New Englad Biolabs	Cat#M0491S
Zymo Clean & Concentrator	Zymo Research	Cat#D4004
SsoAdvanced Universal SYBR Green Supermix	Bio-Rad	Cat#1725270

**Oligonucleotides**		

AC259	5′ gggagcttatcgatgcgtca 3′	
AC260	5′ agtccttcgccagcgttaaa 3′	
AC265	5′ aggcttcaatgtcgccaaga 3′	
AC266	5′ ctctactggcagggcatacg 3′	
AC271	5′ gcggtttcggttcgataagc 3′	
AC272	5′ tactggcacaccttctgctg 3′	
AC273	5′ tgtttgccggatcaagagct 3′	
AC274	5′ cccctgacgagcatcacaaa 3′	

**Recombinant DNA**		

pAC70	This paper	This paper
pAC85	This paper	This paper
pAC76	This paper	This paper
pAC77	This paper	This paper

**Software and algorithms**		

SoftMax Pro 7.1.2	Molecular Devices	https://shop.moleculardevices.com/collections/software-and-instruments/products/softmax-pro-standard
BD Accuri™ C6 Software 1.0.264.21	BD Biosciences	https://www.bdbiosciences.com/en-us/products/software/flowjo-software?tab=flowJo-v11-software
OpenLab software v3.6	Agilent	https://www.agilent.com/en/product/software-informatics/analytical-software-suite/chromatography-data-systems/openlab-cds
CFX Manager 3.1	Bio-Rad	https://www.bio-rad.com/en-us/sku/1845000-cfx-manager-software?ID=1845000
Primer3Plus version: 3.3.0	Untergasser et al. 2012^62^	https://www.primer3plus.com/index.html
PCR miner	Zhao and Fernald, 2005^63^	http://miner.ewindup.cn/miner/
DoriC 10.0	Luo and Gao, 2019^36^	https://tubic.org/doric10/public/index.php
OriV-Finder	Li and Gao, 2025^37^	https://tubic.org/OriV-Finder/OriV-Finder/

**Other**		

Veriti 96-well Thermal Cycler	Thermo Fisher Scientific	Cat#4375305
CFX96 Touch Real-Time PCR Detection System	Bio Rad	Cat#184-5096
8890 GC System	Agilent	Cat#G7110A
Flame Ionization Detector	Agilent	Cat#G3540A
Auto sampler	Agilent	Cat#G4513A
BD C6 Accuri™ flow cytometer	BD Biosciences	Cat#7693A
SpectraMax M2	Molecular Devices	Cat#0112-0102

Deposited Data		

PacBio sequencing data	NCBI BioProject: PRJNA1400610	SAMN54496139
Nanopore sequencing data	NCBI BioProject: PRJNA1400610	SAMN54496138
*Escherichia coli* plate	NCBI BioProject: PRJNA1400610	SAMN54496120
*Escherichia coli* outgrowth	NCBI BioProject: PRJNA1400610	SAMN54496123
*Escherichia coli* liquid time point 1	NCBI BioProject: PRJNA1400610	SAMN54496126
*Escherichia coli* liquid time point 2	NCBI BioProject: PRJNA1400610	SAMN54496129
*Escherichia coli* liquid time point 3	NCBI BioProject: PRJNA1400610	SAMN54496132
*Escherichia coli* liquid time point 4	NCBI BioProject: PRJNA1400610	SAMN54496135
*Pantoea* sp. plate	NCBI BioProject: PRJNA1400610	SAMN54496121
*Pantoea* sp. outgrowth	NCBI BioProject: PRJNA1400610	SAMN54496124
*Pantoea* sp. liquid time point 1	NCBI BioProject: PRJNA1400610	SAMN54496127
*Pantoea* sp. liquid time point 2	NCBI BioProject: PRJNA1400610	SAMN54496130
*Pantoea* sp. liquid time point 3	NCBI BioProject: PRJNA1400610	SAMN54496133
*Pantoea* sp. liquid time point 4	NCBI BioProject: PRJNA1400610	SAMN54496136
*Brevundimonas* sp. plate	NCBI BioProject: PRJNA1400610	SAMN54496122
*Brevundimonas* sp. outgrowth	NCBI BioProject: PRJNA1400610	SAMN54496125
*Brevundimonas* sp. liquid time point 1	NCBI BioProject: PRJNA1400610	SAMN54496128
*Brevundimonas* sp. liquid time point 2	NCBI BioProject: PRJNA1400610	SAMN54496131
*Brevundimonas* sp. liquid time point 3	NCBI BioProject: PRJNA1400610	SAMN54496134
*Brevundimonas* sp. liquid time point 4	NCBI BioProject: PRJNA1400610	SAMN54496137

### Experimental model and study participant details

#### Bacterial strains and growth conditions

The bacterial strains and plasmids used in this study are listed in Data S2. *E. coli* BW25113 was acquired from an LBNL public registry (https://public-registry.jbei.org/).^64^ All other strains listed in Data S2 were isolated from the Oak Ridge Field Research Center (ORFRC); (https://public.ornl.gov/orifc/orfrc3_site.cfm).

Competent cells of *E. coli* TransforMax EC100D *pir*-116 cloning strain and the *E. coli* WM3064 conjugation donor strain were purchased from Lucigen (Biosearch Technologies, Alexandria, MN). *E. coli* 10-β competent cells were purchased from New England BioLabs (NEB, Ipswich, MA, USA). All bacterial strains were grown in liquid and solid medium using a Luria-Bertani (LB) base (Becton Dickinson, Milpitas, CA). Antibiotic concentrations for selection were carbenicillin (carb, 50 μg/mL) and kanamycin (kan, 50 μg/mL). When the *E. coli* WM3064 strain was used, media was supplemented with diaminopimelic acid (DAP) at a concentration of 300 μM.

All non-magic pool plasmids were constructed using standard Gibson cloning methods.^65^ Plasmid DNA isolation was performed using a miniprep plasmid isolation kit according to the manufacturer’s instructions (Qiagen, Redwood City, CA). General microbiological manipulations, PCR amplification, golden gate assembly, cloning, and transformation procedures were described previously.^23^ Enzymes were purchased from New England Biolabs (NEB, Ipswich, MA, USA) and Thermo Fisher Scientific (Waltham, MA, USA). Oligonucleotide primers were obtained from Integrated DNA Technologies (IDT, Coralville, IA, USA). Double strand DNA gene fragments were ordered for synthesis from Genewiz Inc (Azenta Life Sciences, South Planefield, NJ). All plasmids constructed, not including magic pool library construction, were verified by whole plasmid sequencing by Plasmidsaurus Inc (South San Francisco, CA). Unless noted, all reagents and media components were purchased from Sigma-Aldrich (St. Louis, MO, USA).

### Method details

#### Origin of replication prediction

From our earlier plasmidome study, 32 circular plasmid sequences were analyzed using DoriC for prediction of the origin of replication^8^ and the resulting 18 predicted origins of replication were used in the subsequent experimental validation workflow. For this study, DoriC 10.0 was used (details for DoriC 10 are provided at https://tubic.org/doric10/public/index.php).^36^ DoriC has since been updated to Dori 12.0^8^ and a new plasmid focused OriV-finder^37^ tool was also released recently. All 32 scaffolds examined in this study were also analyzed using the OriV-finder tool. Details of OriV-finder are available at https://tubic.org/OriV-Finder/OriV-Finder/. Examined scaffold sequences, original DoriC predictions and the most recent OriV-finder predictions are provided in supplementary data (Data S1).

#### Construction of the kanamycin magic pool origin libraries

Part 1 origin plasmids were synthesized into a pJW52 backbone^65^ by Genscript USA Inc (Piscataway, NJ). Parts 2 (promoter for drug resistance marker) and 3 (drug resistance coding sequence (CDS)) (also in pJW52 backbones) plasmids were constructed previously.^23^ All plasmids containing parts 1, 2, and 3 can be found in Data S1. For all part 1 origins, 100 ng of each plasmid were added to a part 1 mix. This was also done for parts 2 and 3, separately. 100 ng of each mix was then added into the golden gate assembly.^66^ The origin magic pool was constructed by Golden Gate assembly using methods described previously.^23^

The magic pool was built in two dilutions, “1x” and “2x” based on the number of *E. coli* *pir*+ colonies added to 100 mL of LB medium supplemented with DAP and carbenicillin. The magic pool was allowed to grow overnight, followed by a glycerol stock and four plasmid DNA isolations per dilution. An amplification of the barcoded region of each plasmid assembly was performed to check the number of barcodes per assembly. For the magic pool the “1x” dilution was selected due to its barcode coverage relative to the total combination of possible vectors (∼10x). Utilizing the DNA isolated previously, the magic pool was transformed into the *E. coli* WM3064 conjugation donor strain. The entirety of the transformation was then grown in 100 mL of media supplemented with DAP and stored via glycerol stock after an overnight incubation.

The plasmid DNA that was isolated from the *E. coli* *pir*+ cells, and subsequently used to transform into the *E. coli* WM3064 conjugation donor, was sequenced by Plasmidsaurus using long-read sequencing (once with PacBio sequencing technology and once with in-house using Nanopore sequencing) with custom analysis and annotation. Sequencing data was analyzed by searching through each long read and comparing the sequence to a list of parts that were used in the construction. The output of this search was a list of barcodes and the parts (1,2 and 3) associated with the barcode. For us to confidently assign a unique barcode to an origin part, we had to observe at least two unique long-reads that supported this observation. In some cases a single barcode was associated with multiple parts from each grouping of parts (i.e., multiple part 1s can be associated with a single barcode) (see Figure S1 and Data S7 for part 1 barcode-origin associations). These non-unique barcodes were not used for any further analysis.

Part 2.4 showed a 160 bp sequence similarity to the part 4 backbone (promoter driving *bla* gene). This caused the software that analyzed the long read sequencing data to not be able to differentiate between part 2.4 and the backbone. In Figures S2–S4 “unmapped part 2” represents either unmapped part 2s or part 2.4.

#### Magic pool conjugation

The following protocol was adapted from Liu et al.^23^ Recipient strains were streaked out on LB agar for isolated colonies. A single colony of the recipient was used to inoculate a 10 mL volume of LB and grown overnight at 30 °C and shaking at 200 rpm. A 2 mL glycerol stock of the *E. coli* WM3064 donor strain, containing the magic pool library, was inoculated into 50 mL of LB supplemented with carbenicillin and DAP and allowed to grow at 37 °C, while shaking at 200 rpm, for 4 h. One OD of donor cells was pelleted in a microcentrifuge tube and washed twice with LB. One OD of recipient cells was pelleted and washed once with LB. Each conjugation contained a 1:1 OD ratio of donor:recipient cells. A donor pellet and a recipient pellet were resuspended in the same 200 μL of LB and plated onto an LB plate supplemented with DAP. Conjugations were incubated at 30 °C overnight. Cells were harvested from the conjugation plates and resuspended in 4 mL of LB with 15% glycerol. These conjugation stocks were frozen at −80°C.

To determine the number of colonies per mL, a tube of conjugation stock was thawed and serially diluted down to 1x10^−6^. Dilutions 1x10^0^ to 1x10^−4^ were plated on kanamycin selective media while dilutions 1x10^−5^ and 1x10^−6^ were plated on non-selective media to check for transformation efficiency and for survival of the recipient. Colony counts from these plates were used to estimate the volume of conjugation mix would be needed to get ∼5,000 colonies. This volume was then either inoculated directly into liquid selection, and grown for the time course experiments, or plated onto LB supplemented with kanamycin and incubated overnight at 30 °C to form small colonies.

For the liquid selection time course experiment, ∼5,000 CFUs were inoculated into 10 mL of LB supplemented with kanamycin and allowed to grow overnight at 30 °C and shaking at 200 rpm. 1 mL of the culture was pelleted and plasmid DNA was extracted for sequencing. 500 μL of the grown culture was then used to back dilute into 10 mL of fresh LB supplemented with kanamycin. This back dilution happened 3 times resulting in 4 total time points.

For plate-based selection, plates were incubated overnight at 30 °C. Colonies were then harvested and resuspended in LB. The resuspension was diluted to an OD of 3 and pelleted. The plasmid DNA from this pellet was extracted and used for sequencing. With the same resuspension, 50 mL cultures were started at an OD of 0.5. These cultures were incubated overnight at 30 °C and shaken at 200 rpm and pellets were collected the next day for DNA extraction and sequencing.

### Quantification and statistical analysis

#### Analyzing the plasmid fitness within each bacterial strain

For each sample PCR amplification of the DNA barcodes, and BarSeq, was performed as described previously.^23^^,^^67^ On average, 4 million reads were generated per sample. For the plate- and liquid-based experiments, we typically collected ∼5000 colonies per experiment resulting in a read coverage per colony of 800x.

A list of the count of barcodes per sample was generated. Each file, containing barcode sequencing data for one strain’s time point, was filtered to remove all barcode counts that were below 6. After filtering, an average of 98.64% (±0.36%) of the barcode counts remained. The filtered barcodes were then compared to the barcode-part association generated and the resulting data was used to generate all origin-related data, as well as the analysis of each recipient bacterium’s preferred part 2s and part 3s. The barcodes for each time point were also investigated for barcode diversity. This was done by determining the number of barcodes representing each origin present at each time point.

To classify an origin as functional we considered both the plate-based and liquid outgrowth assays, In both instances, we required multiple barcodes associated with a single origin to show high barcode counts. For the purely liquid transfer experiments, we also expected that long-term selection in the presence of kanamycin would lead to instances of some origins “out-performing” others, due potentially to copy number variation in the kanamycin resistance gene. Relatedly, we found that the part 2 promoter sequence had a large impact on driving kanamycin resistance, which could also lead to barcodes associated with functional origins going up or down over multiple transfers. So, for the liquid experiments with multiple transfers we also looked at the behavior and barcode counts of the different origins and their kanamycin resistance cassettes holistically (Figures S2–S4).

#### Colony quantitative PCR

SsoAdvanced Universal SYBR Green Supermix was ordered from BioRad (BioRad Inc, Hercules, CA). All qPCR primers were selected by using Primer3Plus (https://www.primer3plus.com/index.html) using the qPCR settings. Primers for genomic DNA were ordered for gene IAI47_12350 (a glycosyl transferase/lysophospholipid acyltransferase), a gene found only in the *Pantoea* sp. MT58 genome and not on the introduced plasmid. This primer pair amplifies a region of 528 bp. Primers for plasmid amplification were ordered to amplify a region within the origin 6911. This primer pair amplifies a region of 598 bp. Melt curves utilizing all primer pairs show only one amplicon. Plates were run on a CFX96 Real-Time System using Bio-Rad CFX Manager 3.1 software. A fresh single colony of *Pantoea* sp. MT58 with pAC70, *Pantoea* sp. MT58 with pAC75, and *Pantoea* sp. MT58 with pAC77 was picked into 40 μL of Ambion nuclease-free water, separately, and resuspended by pipetting up and down. 20 μL of the resuspended colony was then diluted into 20 μL of water. This was repeated for a total of 3 dilutions (undiluted, 1:2, 1:4, 1:8). 2 μL of each dilution was used for each qPCR reaction, which was performed in triplicate. All colony qPCR data and primers used for all colony qPCR experiments can be found in Data S3. Plasmids will be made available upon request.

The thermocycler protocol and qPCR mastermix were executed as described in David et al.^68^

Copy numbers (CN) were calculated using the following formula^68^ (Equation 1):

(Equation 1) $$\text{CN}=2^{-\left(Ct\;plasmid-Ct\;gDNA\right)}$$

Copy number was then corrected by primer efficiency (E) by the following formula (Equation 2):

(Equation 2) $${\text{CN}}_{\text{adjusted}}=\text{CN}\times \left(E_{\text{gDNA}\;\text{primers}}/E_{\text{plasmid}\;\text{primers}}\right)$$

Primer efficiency was calculated using PCR Miner (http://miner.ewindup.cn/miner/), which calculates the primer efficiency of each well by performing a linear regression of the fluorescence values during the exponential phase of amplification.

The final equation (Equation 3) used to determine the plasmid copy numbers was the following^69^:

(Equation 3) $${\text{CN}}_{\text{adjusted}}=\left(2^{-\left(\text{Ct}\;\text{plasmid}-\text{Ct}\;\text{gDNA}\right)}\right)\times \left(E_{\text{gDNA}\;\text{primers}}\;/E_{\text{plasmid}\;\text{primers}}\right)$$

#### GFP quantification in *Pantoea* sp. MT58

The original magic pool plasmid design utilized a promoter that was non-functional in driving expression of GFP in *Pantoea* sp. MT58. Plasmid pAC80 was constructed combining origin 6911, part 2.4 and part 3.8, as well as an inducible promoter, P_BAD_, to drive expression of the GFP. Plasmid pAC80 for GFP expression was conjugated into *Pantoea* sp. MT58 using standard protocols as described above. The empty vector, pAC87, was constructed by removing the GFP from pAC80.

Both the GFP-containing plasmid and the empty vector were conjugated in *Pantoea* sp. MT58 and successful conjugations were observed through colonies resistant to kanamycin. Three colonies of *Pantoea* sp. MT58 containing pAC80 and three colonies of *Pantoea* sp. MT58 containing the empty vector were each inoculated into a 24 deep well plate containing 3 mL of LB with kanamycin and 0.3% (w/v) arabinose and grown overnight at 30 °C while shaking at 200 rpm. After growing overnight, each culture was split into three technical replicates and back diluted (150 μL into 3 mL) in 3 mL of LB with kanamycin and 0.3% (w/v) arabinose for another overnight growth.

1 μL was sampled from the dense overnight culture and added to 99 μL of phosphate buffered saline (PBS) in a 96 well flat bottom plate. GFP was quantified by using a BD C6 Accuri flow cytometer by reading 30,000 events using a medium flow rate (35 μL/min, core size = 16 μm). One wash cycle was performed in between each well. Raw data can be found in Data S4. Representative flow cytometry data can be seen in Figure S6.

#### Indigoidine production

Indigoidine production plasmid pAC75 and empty vector pAC87 were conjugated into *Pantoea* sp. MT58 using standard protocols as described above. A single colony of each strain was inoculated into 5 mL of LB with kanamycin and grown overnight at 30 °C while shaking at 200 rpm. The following day, 300 μL of the seed culture was back diluted into a 24 deep well plate, in triplicate, containing 3 mL of LB with kanamycin and 0.3% (w/v) arabinose. This plate was incubated in a shaker at 30 °C and shaking at 900 rpm. After 24 h, 200 μL of each sample was taken from the plate and spun down at 14,000 rpm for 5 min. The 24 DWP was then put back into the shaker for another 24 h in which the final time point would be taken. Extraction occurred by removing the supernatant of spun down cultures, resuspending the pellet in 500 μL of DMSO and shaking at 3000 rpm for 20 min. Due to the high absorbance of the sample, 20 μL of the extracted material was diluted into 80 μL DMSO and then put into a clear bottom 96 well plate and absorbance was measured at 612 nm (Data S5).

#### Isoprenol production

Experiments to determine isoprenol toxicity, catabolism and production were performed using minimal salt (M9) medium composed of 1x M9 salts (6.8 g/L Na_2_HPO_4_, 3 g/L KH_2_PO_4_, 0.5 g/L NaCl), 1% (w/v) glucose, 10 mM (NH_4_)_2_SO_4_, 2 mM MgSO_4_, 0.1 mM CaCl_2_ and trace metal solution (500 μL per 1 L medium, Product No. 1001, Teknova Inc, Hollister, CA).^49^^,^^70^ All inoculations were grown at 30 °C and shaken at 200 rpm. Isoprenol production plasmid pAC104 was electroporated into *Pantoea* sp. MT58. Electrocompetent cells were prepared by spinning down 2.25 mL of an overnight culture at stationary phase at 8000 rpm for 3 min. Supernatant was removed and the pellet was washed with 750 μL of 10% glycerol three times, centrifuging at 8000 rpm for 3 min each wash. Washed pellet was resuspended in 250 μL of 10% glycerol. 50 μL of cells was mixed with 50 ng of DNA and electroporated at 2.5 kV/cm in a 1 cm cuvette. Cells were recovered in SOC and shaking at 900 rpm for 1 h. 200 μL of cells were plated on LB supplemented with kanamycin and grown overnight.

To determine isoprenol toxicity and catabolism, three single colonies of *Pantoea* sp. MT58 were inoculated in 5 mL LB in glass culture tubes and allowed to grow overnight at 30 °C and shaking at 200 rpm. Cultures were then back diluted in 5 mL M9 with 1% glucose in glass culture tubes and allowed to grow overnight at 30°C and shaking at 200 rpm. This overnight culture was then used as an inoculum for further experiments as follows.

For the isoprenol consumption assay, cultures were then inoculated again in M9 (either with or without 1% glucose) either with, or without, 1 g/L isoprenol in a 24 deep well plate at an OD of 0.01, which were sealed and incubated at 30 °C with agitation in a microplate reader for 24 h.

For the isoprenol toxicity assay, cultures were then inoculated again in M9 with 1% glucose and varying concentrations of isoprenol in a 24 deep well plate at an OD of 0.01, which were sealed and incubated at 30 °C with agitation in a microplate reader for 24 h.

All plates were sealed with a semi-permeable film (Breathe Easy Film, USA Scientific, Ocala, FL) and incubated in a microplate reader (Molecule Devices M2 Plate Reader, San Jose, CA). Readings were taken every 15 min with continuous agitation at 30 °C in between readings (Figure S8).

For isoprenol production, three single colonies of *Pantoea* sp. MT58 were inoculated in 5 mL of LB supplemented with kanamycin in glass culture tubes and allowed to grow overnight at 30 °C while shaking at 200 rpm. Cultures were then back diluted in 5 mL M9 supplemented with 1% glucose and kanamycin in glass culture tubes and allowed to grow overnight at 30 °C while shaking at 200 rpm. Cultures were then back diluted in 5 mL of M9 supplemented with 1% glucose, kanamycin and either 0.2% (w/v), 1% (w/v) or no arabinose in glass culture tubes and were allowed to grow overnight at 30 °C while shaking at 200 rpm. At 24, 48, and 72 h time points samples were collected to measure cell density (Figure S10) and isoprenol according to Banerjee et al.^49^ Samples were mixed with equal volumes of ethyl acetate and vortexed at max speed for 15 min. Samples were then centrifuged and 80 μL of the upper organic layer was used for analysis using Gas Chromatography- Flame Ionization Detector (GC-FID). 80 μL of the ethyl acetate layer was transferred to a GC vial containing an insert, and 1 μL was analyzed using an Agilent Technologies GC 8890 system (USA) with a FID and a DB-WAX capillary column (15 m × 0.25 mm × 0.25 μm, Agilent Technologies, USA) for quantification. Helium served as the carrier gas at a flow rate of 2.2 mL/min, and the injection volume was 1 μL in splitless mode. The injector and detector temperatures were set to 250 °C and 300 °C, respectively. The GC oven followed a programmed temperature gradient: an initial hold at 40 °C, a first ramping to 100 °C at 15 °C/min and then to 230 °C at 30 °C/min followed by a final hold at 230 °C for 1 min. Data collection and analysis were performed using OpenLab software (Agilent Technologies, USA). Analytical grade standards obtained from Sigma-Aldrich (St. Louis, MO, USA) were used to determine isoprenol concentration.
