# Supplementary material for: Origin of replication discovery for environmentally isolated Pantoea strain enables expression of heterologous proteins, pathways and products
Source: iScience. 2026 Feb 17;29(3):115031. doi: 10.1016/j.isci.2026.115031 (PMC12969353; doi:10.1016/j.isci.2026.115031)
Supplement: Document S1. Figures S1–S10 [file mmc1.pdf]

iScience, Volume *29*

Supplemental information

Origin of replication discovery for environmentally isolated *Pantoea* strain enables expression of heterologous proteins, pathways and products

Alex Codik, Ankita Kothari, Hualan Liu, Benjamin L. Weinberg, Trenton K. Owens, Aparajitha Srinivasan, Alex Rivier, Thomas Eng, Adam P. Arkin, Adam M. Deutschbauer, and Aindrila Mukhopadhyay

Supplementary Figures:

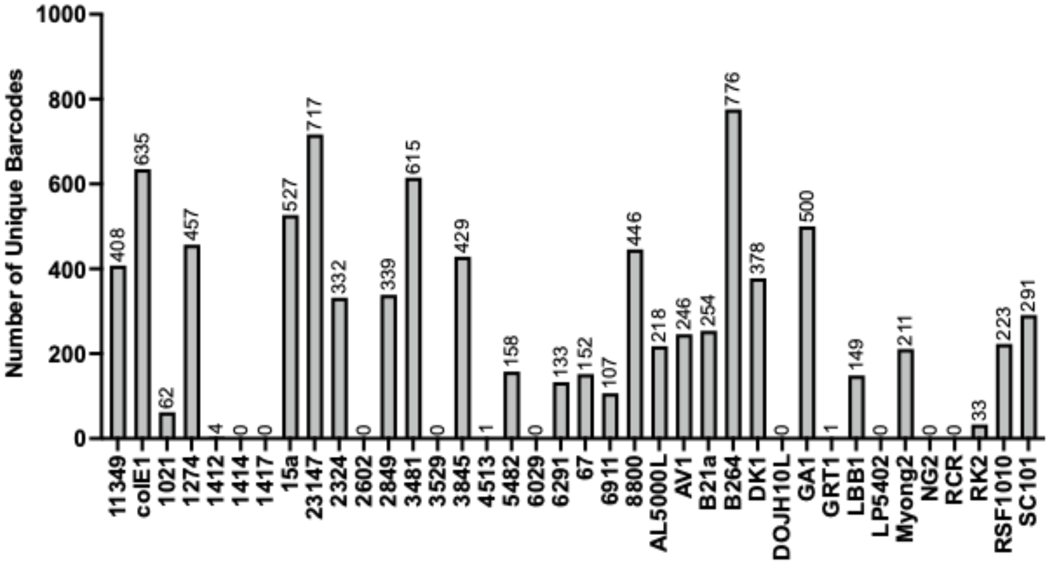

Figure S1. Number of unique barcodes per origin present in the magic pool.

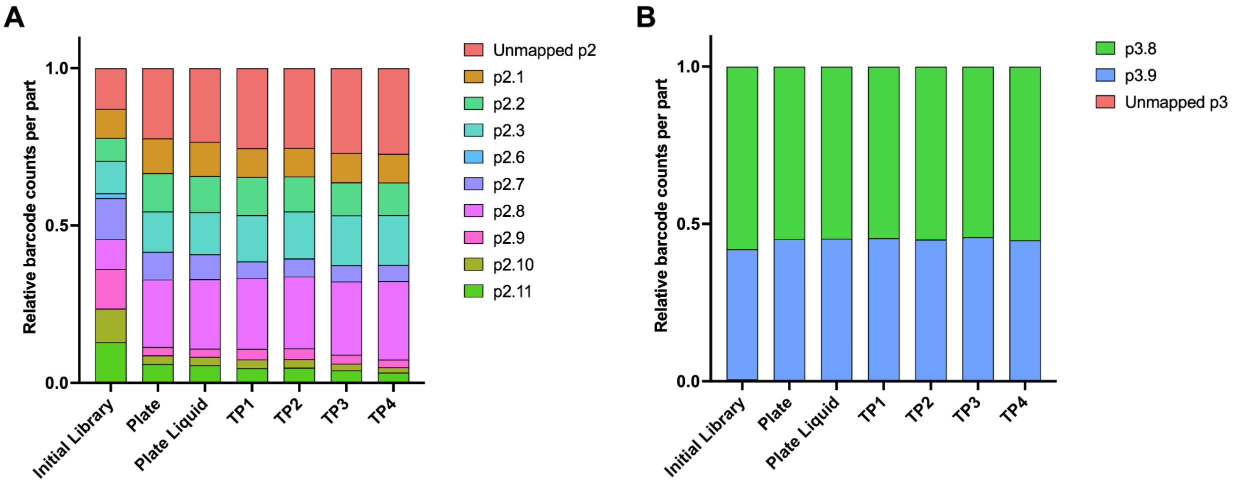

Figure S2. Breakdown of part 2 and 3 variants in *E. coli* BW25113.

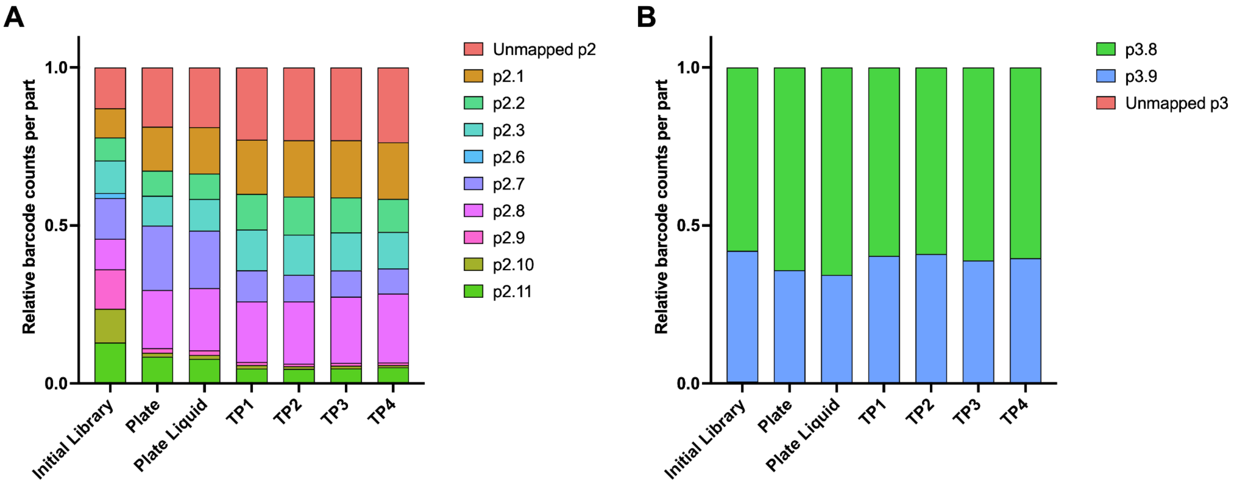

Figure S3. Breakdown of part 2 and 3 variants in *Pantoea* sp. MT58.

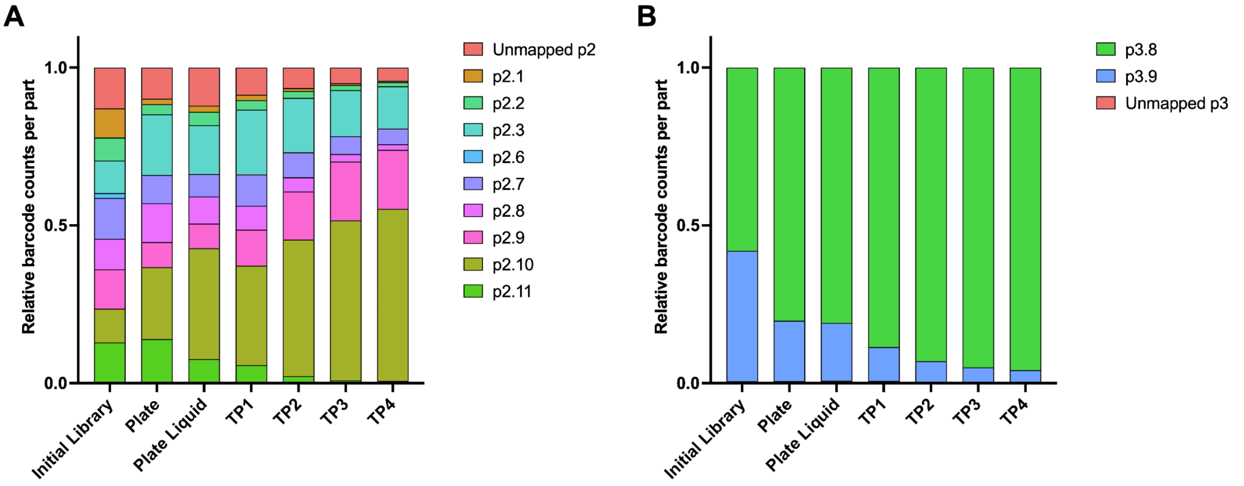

Figure S4. Breakdown of part 2 and 3 variants in *Brevundimonas* sp. GW460-12-10-14-LB2.

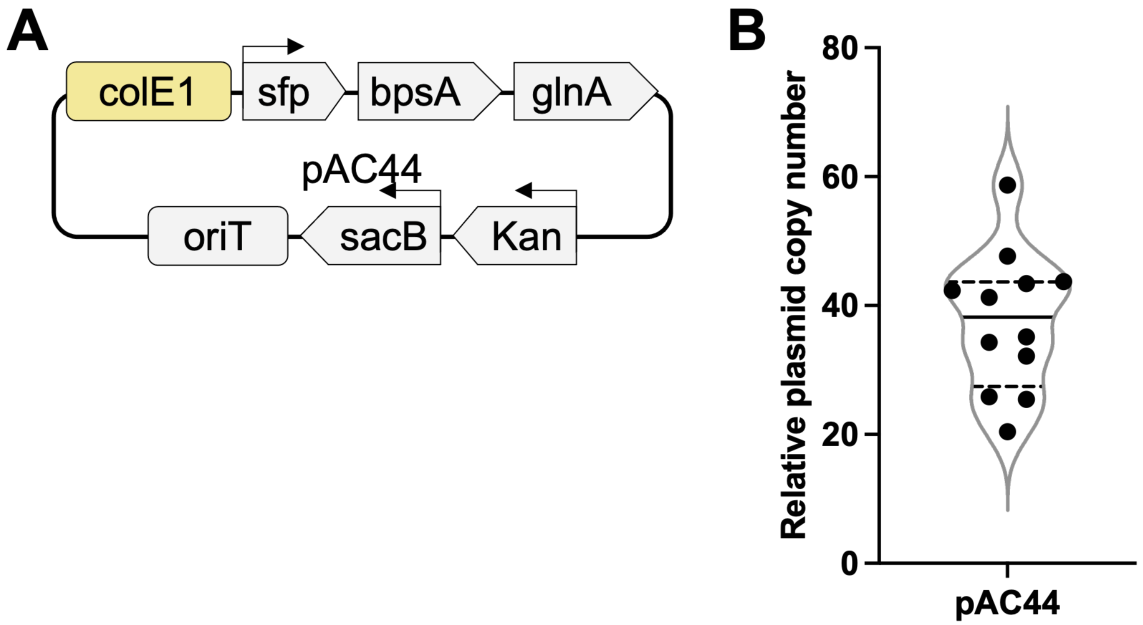

Figure S5. Validation of colony qPCR method through testing known colE1 origin. A) Plasmid map of plasmid used to generate qPCR data in (B). B) Violin plot of plasmid copy number. A solid vertical line represents the data median. Vertical dashed lines represent quartiles.

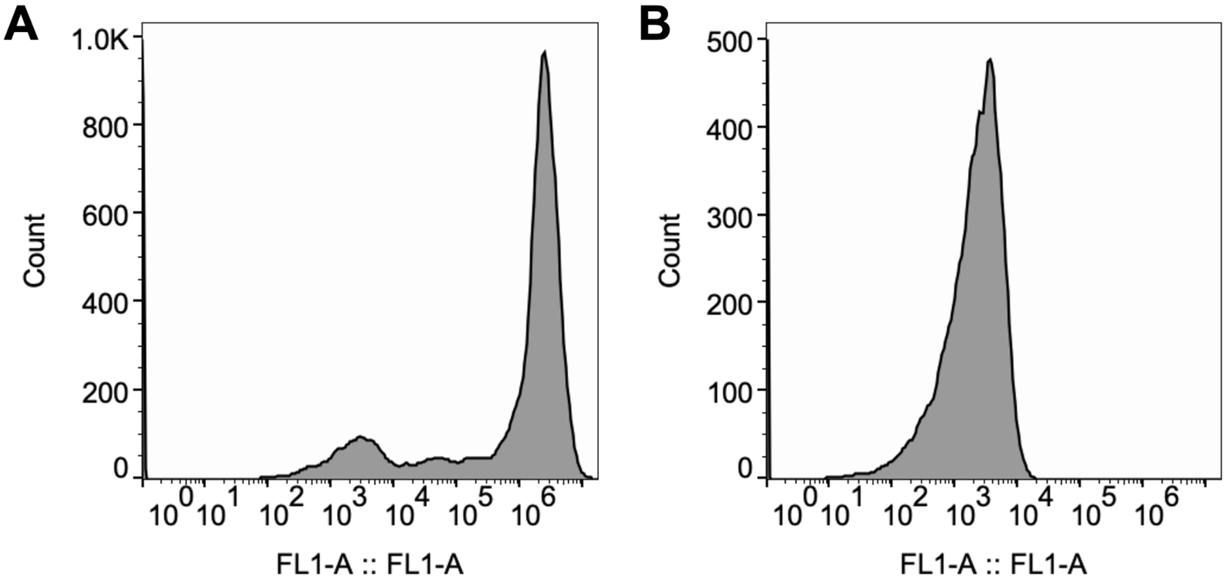

Figure S6. Flow cytometry GFP expression data. A) Representative fluorescence data from GFP expressing *Pantoea* sp. MT58 cells. B) Representative fluorescence data from *Pantoea* sp. MT58 cells without the GFP-expressing plasmid.

A

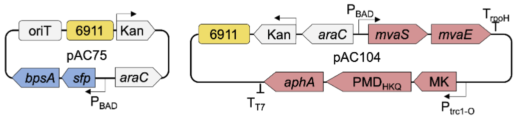

B

Indigoidine pathway (pAC75)		Isoprenol pathway (pAC104)	
**Gene name**	**Gene Description**	**Gene name**	**Gene Description**
bpsA	Blue pigment synthetase A	*mvaS*	hydroxymethylglutaryl-CoA synthase
sfp	PPTase	*mvaE*	acetyl-CoA acetyltransferase
		MK	mevalonate kinase
		PMD_{HKQ}	phosphomevalonate decarboxylase
		aphA	Aminoglycoside 3'-phosphotransferase

C

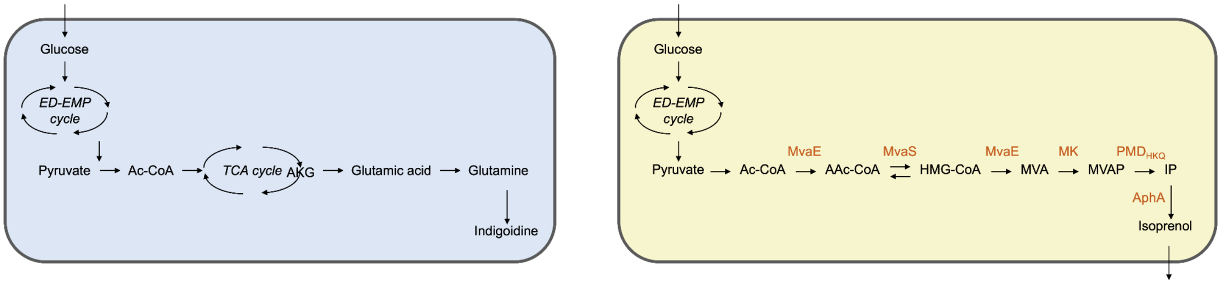

Figure S7. Indigoidine and isoprenol pathway breakdowns. A) Plasmid maps of plasmids used to express indigoidine (left panel) and isoprenol (right panel) in *Pantoea* sp. MT58. B) Gene name and description of genes used in each plasmid for expression of indigoidine and isoprenol. C) Representative cell map and metabolisms to produce indigoidine (left panel) and isoprenol (right panel).

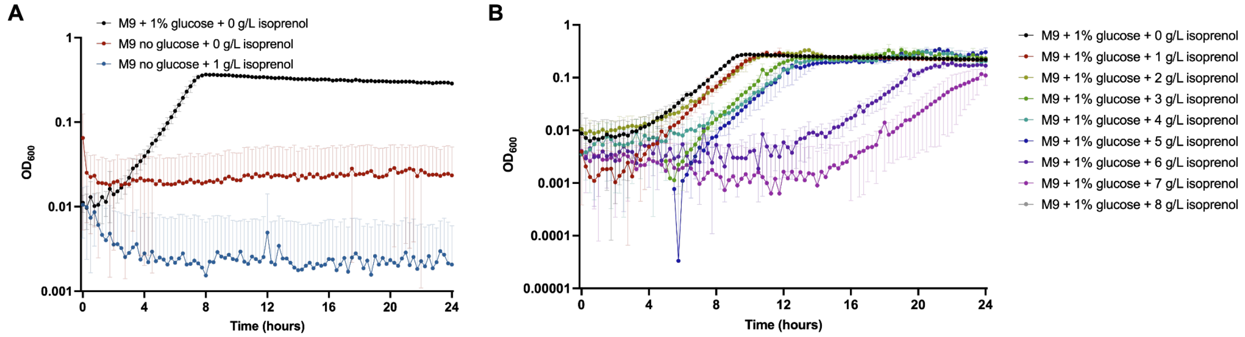

Figure S8. Isoprenol degradation and tolerance profile in *Pantoea* sp. MT58 A) Determination of *Pantoea* sp. MT58's ability to consume isoprenol as a sole carbon source. B) *Pantoea* sp. MT58's growth response to varying concentrations of isoprenol supplemented with M9 1% glucose minimal medium. The data for M9 + 1% glucose + 8 g/L isoprenol, when corrected against the blank, resulted in negative values, and are indicative of a lack of growth. All error bars represent standard deviation.

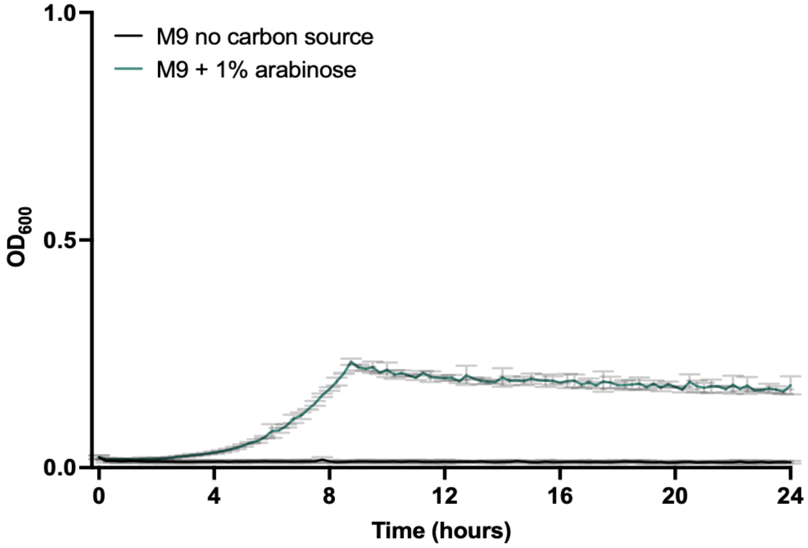

Figure S9. *Pantoea* sp. MT58 growth on arabinose as a sole carbon source. Cells grown in a 96 well plate. All error bars represent standard deviation. N = 3

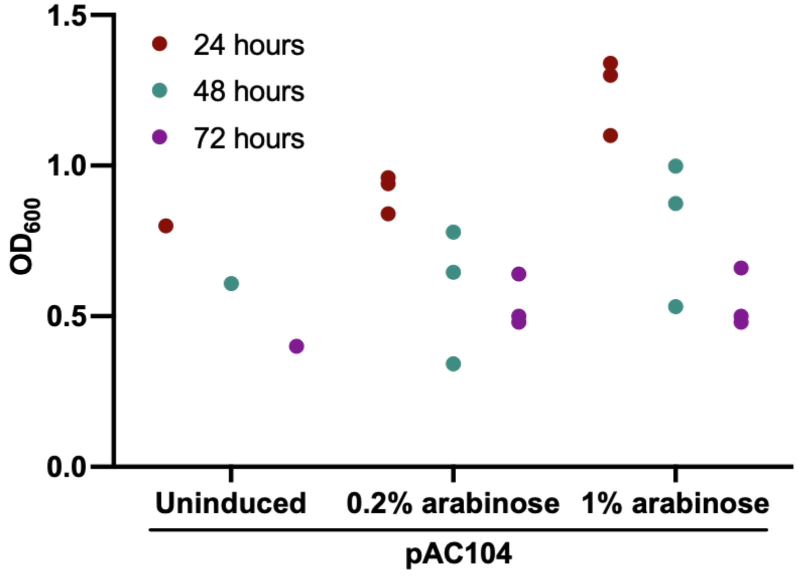

Figure S10. Optical density measurements for isoprenol production run.

Supplemental Data files provided separately:

- Parts and their sequences - Data_S1.xlsx
- Strain and plasmid table - Data_S2.xlsx
- qPCR raw data/primers used - Data_S3.xlsx
- GFP expression - Data_S4.xlsx
- Indigoidine absorbance data - Data_S5.xlsx
- Isoprenol expression data - Data_S6.xlsx
- Barcode-part combinations - Data_S7.xlsx
